# Mealworm Oil (MWO) Enhances Wound Healing Potential through the Activation of Fibroblast and Endothelial Cells

**DOI:** 10.3390/molecules26040779

**Published:** 2021-02-03

**Authors:** Joung-Hee Kim, Eun-Yeong Kim, Kyu Jin Chung, Jung-Hee Lee, Hee-Jung Choi, Tae-Wook Chung, Keuk-Jun Kim

**Affiliations:** 1 Department of Biomedical Laboratory Science, TaeKyeung University, 65, Danbuk 1-gil, Jain-myeon, Gyeongsan-si, Gyeongsangbuk-do 38547, Korea; k9j1h@naver.com; 2 APROGEN, Inc., 545, Dunchon-daero, Jungwon-gu, Seongnam-si, Gyeonggi-do 13215, Korea; eylove0822@hanmail.net; 3 Department of Plastic and Reconstructive Surgery, Yeungnam University College of Medicine, 170, Hyeonchung-ro, Nam-gu, Daegu 42415, Korea; guzy7@hanmail.net; 4 JIN BioCell Co., Ltd., #118-119, National Clinical Research Center for Korean Medicine, Pusan National University Korean Medicine Hospital, 20 Geumo-ro, Mulgeum-eup, Yangsan-si, Gyeongsangnam-do 50612, Korea; desire1213@naver.com (J.-H.L.); heejung0917@gmail.com (H.-J.C.)

**Keywords:** mealworm oil, wound healing, fibroblasts, endothelial cells, migration

## Abstract

Mealworm and mealworm oil (MWO) have been reported to affect antioxidant, anti-coagulation, anti-adipogenic and anti-inflammatory activities. However, the function of MWO in wound healing is still unclear. In this study, we found that MWO induced the migration of fibroblast cells and mRNA expressions of wound healing factors such as alpha-smooth muscle actin (α-SMA), collagen-1 (COL-1) and vascular endothelial growth factor (VEGF) in fibroblast cells. The tube formation and migration of endothelial cells were promoted through the activation of VEGF/VEGF receptor-2 (VEGFR-2)-mediated downstream signals including AKT, extracellular signal-regulated kinase (ERK) and p38 by MWO-stimulated fibroblasts for angiogenesis. Moreover, we confirmed that MWO promoted skin wound repair by collagen synthesis, re-epithelialization and angiogenesis in an in vivo excisional wound model. These results demonstrate that MWO might have potential as a therapeutic agent for the treatment of skin wounds.

## 1. Introduction

The skin of the human body functions in the maintenance of body temperature and as a barrier, protecting it from harmful elements such as ultra-violet (UV), pathogenic microorganisms and toxic materials in the external environment [1]. If an injury has occurred on the skin, acute wound healing including a very complex multistep process such as inflammation, granulation, re-epithelization, angiogenesis and tissue remodeling is needed for protection of the body against severe pathogens [2]. Briefly, in the inflammatory phase, immune cells are attracted to the wound area to remove the damaged tissue and pathogens. In the proliferative phase, the migration and proliferation of fibroblasts to the wound lesion results in the induction of granulation tissue formation by secreting new extracellular matrix (ECM). In the re-epithelialization process, keratinocytes from the edges of the wound also migrate toward the center and proliferate to form new tissue. Next, angiogenesis is a pivotal process to establish a new blood supply to the newly formed tissue. As a process of tissue remodeling, to provide more tensile strength, collagen bundles are increased and more organized. Moreover, the transformation of fibroblasts into myofibroblasts promotes wound contraction. Finally, the wound healing process is completed by removal of excess ECM [3,4].

The mealworm (*Tenebrio molitor*), is used commercially as an edible insect for animal feed and also as a protein-rich food source for human consumption [5]. Furthermore, it has been reported that mealworm oil, which is similar to vegetable oil, contains bioactive nutrients such as unsaturated fatty acids and γ-tocopherol [5,6]. Recent papers show that the mealworm has effects on the antioxidant, anti-coagulation, anti-adipogenic and anti-inflammatory activities [6,7,8]. The Kim et al. group suggests that ingestion of mealworms as a food with a high content of protein can effectively improve the nutritional status and enhance the immune function by clinical application, since protein intake is important for the recovery of the immune system, physical strength and wound healing after surgery [9]. However, based on scientific literature, there have been no reports that explain the function of mealworm oil in wound repair to date.

In the present study, we investigated the effects of mealworm oil (MWO) on the proliferation and migration of fibroblasts and the expressions of wound healing-related genes in fibroblast cells during the wound healing process. Furthermore, the activation of endothelial cells mediated by MWO-stimulated fibroblasts for angiogenesis was tested. We also evaluated the wound repair activity of MWO in an in vivo excisional wound model using rats.

## 2. Results

### 2.1. Effect of MWO on Cell Viability

**Figure 1:**
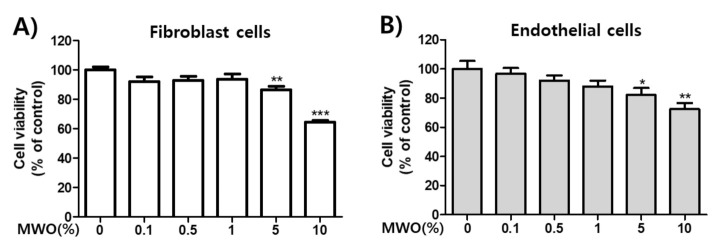
Effect of mealworm oil (MWO) on cell viability in fibroblast and endothelial cells. (**A**) Fibroblast or (**B**) endothelial cells were treated with the indicated concentrations of MWO for 24 h. The viabilities of cells were evaluated by MTT assay. The values are shown as means ± SD. * *p* < 0.05, ** *p* < 0.01 and *** *p* < 0.001 compared to the control.

We confirmed cytotoxicity of MWO to HUVECs and NIH-3T3 cells. The cells were treated with the indicated concentrations of MWO for 24 h and then measured using an MTT assay. MWO treatment showed no significant cytotoxic effects on both the cells at concentrations up to 1% (Figure 1). Thus, we used the 1% and less than 1% concentration of MWO for subsequent experiments.

### 2.2. Regulation of mRNA Expressions of Genes Related to Wound Healing by MWO in Fibroblasts

**Figure 2:**
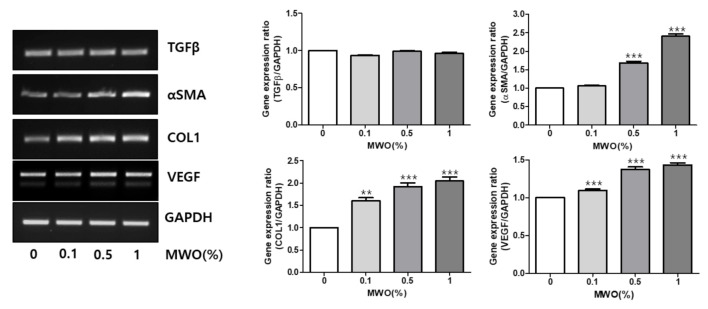
Expression of genes related to wound healing by MWO in fibroblasts. Fibroblast cells were treated with the indicated concentrations of MWO for 24 h. Total RNA was isolated from NIH-3T3 cells. RT–PCR was performed to analyze the mRNA expression of each gene using gene-specific primers, as described in Table 1. GAPDH was used as an internal control. The values are shown as means ± SD. ** *p* < 0.01 and *** *p* < 0.001 compared to the control.

In the wound healing process, it is important that fibroblasts proliferate and migrate [4,10] for extracellular matrix (ECM) formation [11,12]. We checked mRNA levels of *TGF-β1*, which enhances fibroblast proliferation and collagen deposition [13]; α-SMA, as a fibroblast-specific marker playing an important role in the remodeling phase of wound healing [14]; COL-1, as a major protein of the ECM [15]; and VEGF, which is involved in angiogenesis as a major factor required to maintain a critical role in wound healing. After treatment of NIH-3T3 cells with the indicated concentrations of MWO, mRNA levels of *TGF-β1, α-SMA, COL-1, VEGF* and *GAPDH* were estimated by RT-PCR. As shown in Figure 2, the mRNA expression levels of *COL-1, α-SMA* and *VEGF* were increased by MWO. These results suggest that MWO induces gene expression related to wound healing in fibroblasts.

### 2.3. The Enhanced Migration of Fibroblasts by MWO

**Figure 3:**
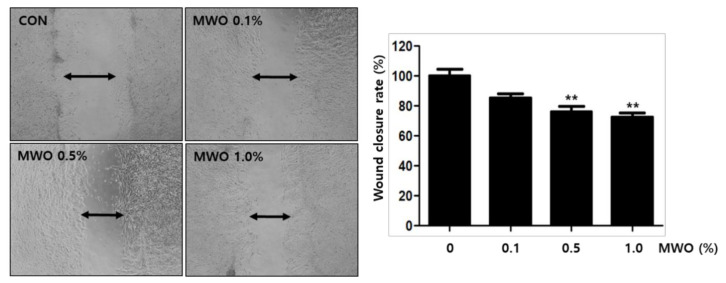
Effect of MWO on migration of fibroblast cells in a wound scratch assay. The culture well was fully filled with fibroblast cells. Wound gaps were then created by scratching with a scraper. The cells were treated with the indicated concentration of MWO for 24 h. The migration of fibroblast cells was observed and photographed with a Nikon light microscope. The quantitative evaluation of wound closure in the wound scratch assay was measured by the distance between the two front lines with the majority of migrated fibroblast cells. The values are shown as means ± SD. ** *p* < 0.01 compared to the control.

To form granulation tissue, it is necessary that fibroblasts migrate to the wound area during the proliferative phase [10,16]. Thus, we performed scratch wound assays, as described in a previous report, to investigate whether MWO affects the migration of fibroblast cells [17]. Fibroblast cells with wound gaps were incubated with the indicated concentration of MWO for 24 h. The wound gaps of the fibroblasts narrowed by MWO treatment in a dose-dependent manner. This result showed that the migration of fibroblast cells was induced by MWO (Figure 3).

### 2.4. The Induction of Capillary-Like Tube Formation and Migration through VEGFR-2 Activation of Endothelial Cells by MWO-Stimulated Fibroblasts

**Figure 4:**
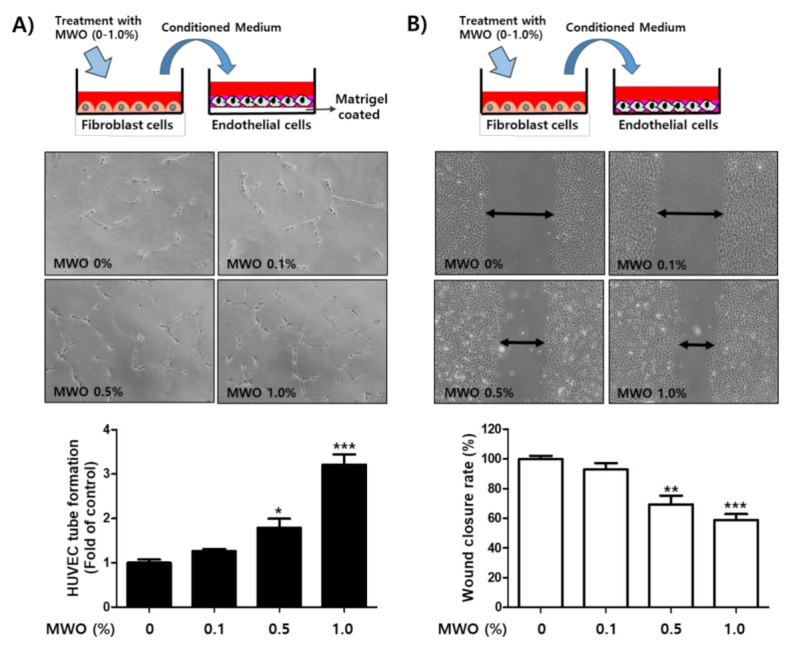

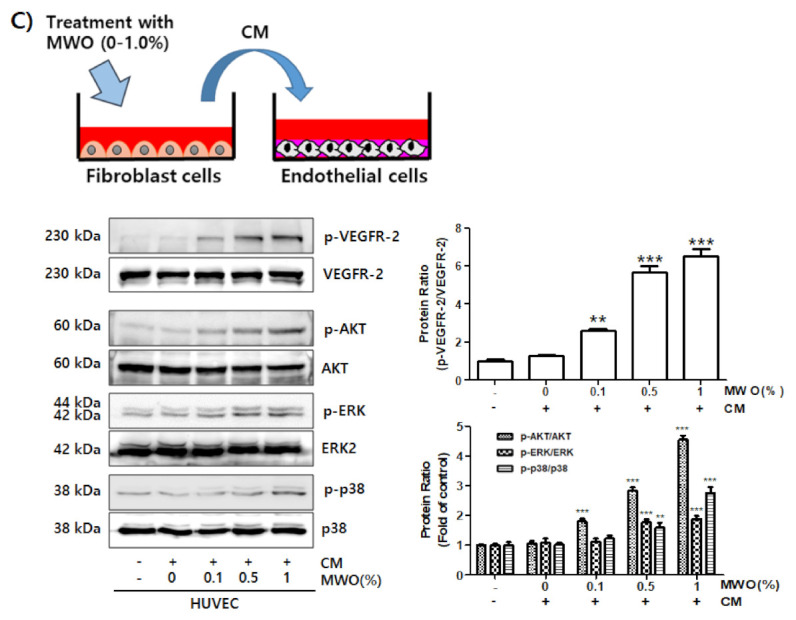
Effect of conditioned media (CM) from MWO-stimulated fibroblasts on tube formation and migration through VEGFR-2 activation of endothelial cells. (**A**) Endothelial cells were cultured on Matrigel-coated plates with the CM harvested from fibroblast cells treated with or without MWO. After 12 h of incubation, capillary-like tube formation was photographed with a Nikon light microscope. Quantitative summary of tube formation was performed by manual quantification of closed tubes. The values are shown as means ± SD. * *p* < 0.05 and *** *p* < 0.001 compared to the control. (**B**) The culture well was fully filled with endothelial cells. Wound gaps were then created by scratching with a scraper. The cells were treated with the CM harvested from fibroblast cells treated with or without MWO for 24 h. The migration of endothelial cells was observed and photographed with a Nikon light microscope. The quantitative evaluation of wound closure in the wound scratch assay was measured by the distance between the two front lines with the majority of migrated endothelial cells. The values are shown as means ± SD. ** *p* < 0.01 and *** *p* < 0.001 compared to the control. (**C**) The endothelial cells were treated with the CM harvested from fibroblast cells treated with or without MWO for 24 h. The total protein extracts harvested from the cells were analyzed by Western blot analysis with antibodies against VEGFR-2, phospho-VEGFR-2, AKT, phospho-_AKT, ERK1/2, phospho-ERK1/2, p38 and phospho-p38. Phosphorylated VEGFR2, AKT, ERK and p38 were standardized relative to their respective total proteins. The values are shown as means ± SD. ** *p* < 0.01 and *** *p* < 0.001 compared to the control.

Angiogenesis is an essential process for the supply of nutrients and oxygen to newly formed tissue during successive proliferative phases of the wound healing process. Furthermore, VEGF, a secretory angiogenic factor, is produced from fibroblasts during wound healing. The increased VEGF expression stimulates endothelial cells to invade a basement membrane for *epidermal* regeneration [15,18,19,20]. In our previous data, VEGF expression was increased by MWO treatment of fibroblast cells (Figure 2), although the expression of VEGF in endothelial cells was not directly changed by MWO treatment (data not shown). Thus, we checked whether CM harvested from fibroblast cells activates endothelial cells for angiogenesis. The medium harvested from MWO-treated fibroblast cells resulted in enhanced tube formation and migration of endothelial cells in a dose-dependent manner (Figure 4A,B). In addition, cultured media from MWO-treated fibroblasts induced not only the activation of VEGFR-2, but also downstream-signaling pathways of VEGFR-2 including ERK, AKT and p38 related to the proliferation, migration and tube formation of VEGF-mediated endothelial cells (Figure 4C). These results suggest that MWO induces angiogenesis for wound healing through the stimulation of fibroblast-mediated endothelial cells.

### 2.5. Effect of MWO on Skin Wound Healing in Rats

**Figure 5:**
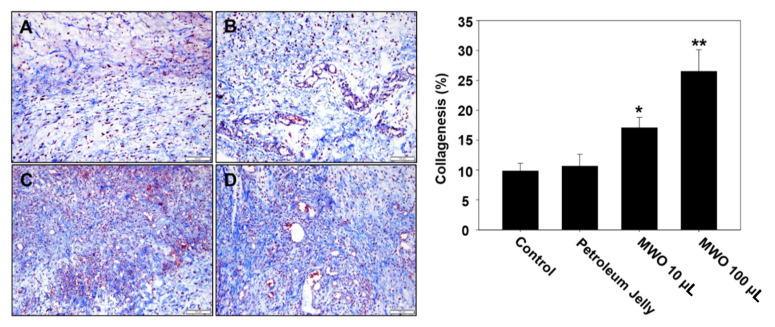
Effect of MWO on the collagen deposition in skin wounds of rats. The tissues harvested from the skin wound area of rats treated with or without MWO were used in Masson’s trichrome staining for collagenesis. Masson’s trichrome staining between skin matrix is represented by the blue color (magnification ×200). The values are shown as means ± SD. * *p* < 0.05 and ** *p* < 0.01 compared to the control. (**A**) The skin wound area untreated with MWO. (**B**) Treated with petroleum jelly as a negative control. (**C**) Treated with 10 μL MWO. (**D**) Treated with 100 μL MWO.

**Figure 6:**
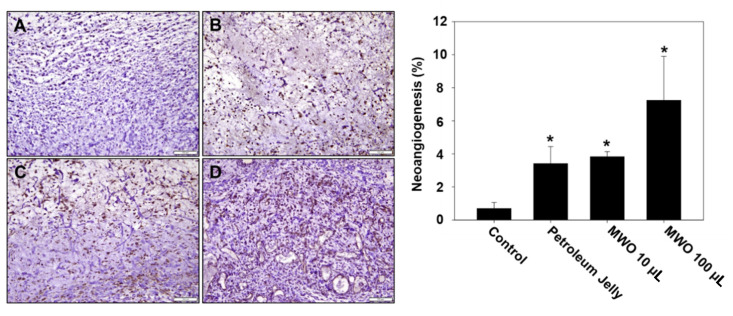
Effect of MWO on angiogenesis in skin wounds of rats. The tissues harvested from the skin wound area of the rats treated with or without MWO were used in immunohistochemistry staining with anti-CD31 to detect neo-vessels. Immunohistochemistry staining of endothelial cells with anti-CD31 is represented by the brown color (magnification ×200). The values are shown as means ± SD. * *p* < 0.05 compared to the control. (**A**) The skin wound area untreated with MWO. (**B**) Treated with petroleum jelly as a negative control. (**C**) Treated with 10 μL MWO. (**D**) Treated with 100 μL MWO.

**Figure 7:**
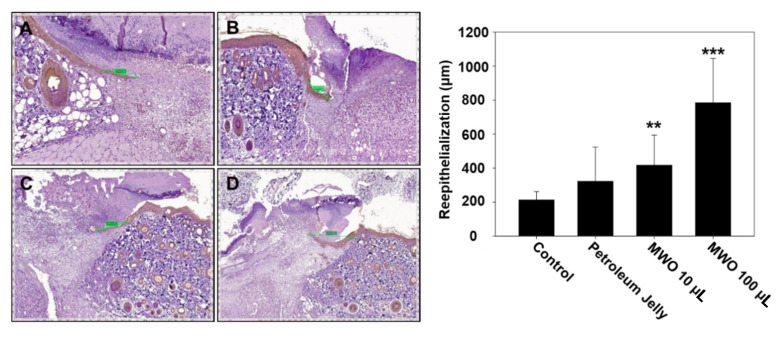
Effect of MWO on re-epithelialization in skin wounds of rats. The tissues harvested from the skin wound area of rats treated with or without MWO were used in immunohistochemistry staining with anti-pan-cytokeratin for re-epithelialization. Immunohistochemistry staining with anti-pan-cytokeratin is represented by the brown color (magnification ×12.5). The values are shown as means ± SD. ** *p* < 0.01 and *** *p* < 0.001 compared to the control. (**A**) The skin wound area untreated with MWO. (**B**) Treated with petroleum jelly as a negative control. (**C**) Treated with 10 μL MWO. (**D**) Treated with 100 μL MWO.

Based on our in vitro results, MWO resulted in the induction of the angiogenic effect and myofibroblast activity, indicating the facilitation of wound healing. To test the effects of MWO on skin wound healing, we checked the healing process using SD rats wounded on the dorsa skin of their backs. Three days after wounds on the skin of the rats were treated with or without MWO, wound repair was observed. As shown in Figure 5, the intensity of collagen deposition in the wounds treated with MWO was increased compared to control as investigated by H & E and Masson trichrome staining. Additionally, collagen fibers were rearranged in a more organized fashion in the MWO-treated wounds compared to the control. Moreover, the expression of CD31 as a marker of vascular endothelial cells for angiogenesis was significantly increased in the MWO-treated wounds compared to control as determined by Immunohistochemistry against CD31 antibodies (Figure 6). Furthermore, the length of regenerated epithelium by treatment with MWO was markedly increased compared to the control as tested by immunohistochemical staining against pan-cytokeratin (Figure 7). These results indicate that MWO could promote collagen deposition along with the remodeling and formation of blood vessels for the process of wound healing in the in vivo experimental animal model.

## 3. Discussion

Skin trauma is a frequently occurring injury caused by various factors. Thus, many researchers are making investigations into finding effective drugs with low side effects for skin wound repair. It has been reported that mealworm and mealworm oil affect antioxidant, anti-coagulation, anti-adipogenic and anti-inflammatory activities. However, the function of MWO for skin wound healing has not been reported. In the present study, our results indicated that MWO could improve skin wound healing by regulating collagen deposition, epithelialization and angiogenesis.

Wound healing is a very complex process including inflammation, granulation, re-epithelization, angiogenesis and tissue remodeling through the interaction among various repair cells, growth factors and skin matrix [1,2]. Fibroblasts play important roles in skin wound repair to re-build the physical barrier through the migration, proliferation and secretion of a large number of collagen fibers and matrix components [21,22]. Thus, we investigated the function of MWO to modulate the activity of fibroblasts for wound healing. As shown in Figure 2, the expression of α-SMA as a myofibroblast phenotype was induced by MWO for wound contraction. Furthermore, MWO increased the gene expression of collagen-1 as a key extracellular matrix of the skin for wound repair. In animal models, MWO markedly affected the intensity of collagen deposition in the wound sites (Figure 5). Although MWO had no effect on the expression of TGF-β1 as an essential factor in wound healing, the migration of fibroblast cells, as one of the important steps, was significantly induced by MWO. These results indicate that MWO might positively regulate the activity of fibroblasts, one of the essential cell types in wound healing.

Angiogenesis is an important factor in the wound repair process for restoration of blood flow to damaged tissues, which supports the growth and function of reparative cells by supplying oxygen and nutrients through newly formed blood vessels. During wound healing, VEGF is produced by a variety of cell types including fibroblasts and is a multifunctional growth factor acting on vascular endothelial cells. Moreover, functions of VEGF are not only angiogenesis but have also recently shown epithelialization and collagen deposition on the wound healing cascade [10,23,24,25]. Interestingly, our data show that MWO resulted in the induction of VEGF expression in the fibroblast cells (Figure 2).

VEGF and its receptor VEGFR-2 are required for the formation of new capillaries from pre-existing blood vessels for angiogenesis. In addition, VEGF-mediated VEGFR-2 activation is closely associated with angiogenic activities such as proliferation, permeability, sprouting, migration, survival and tube formation of endothelial cells via the activation of VEGFR-2 and its downstream signal pathways, such as phosphoinositide 3-kinase (PI-3K)/AKT, protein kinase C (PKC)-dependent rapidly accelerated fibrosarcoma (RAF)/mitogen-activated protein kinase kinase (MEK)/ERK, the tyrosine phosphorylation of vascular endothelial (VE)-cadherin and focal adhesion kinase (FAK) [23,24,25]. As shown in Figure 4, the secretion of VEGF in MWO-treated fibroblast cells affected VEGFR-2 activation on the endothelial cells. Furthermore, VEGF-mediated VEGFR-2 activation by MWO had effects on the tube formation and migration of endothelial cells for angiogenesis, resulting in the activation of VEGFR-2 downstream pathways including AKT, p38 and ERK (Figure 4). As shown in Figure 6, MWO clearly induced the expression of CD31, a marker protein of endothelial cells for angiogenesis at the sites of wounds on the dorsa skin at the back of the rats.

Re-epithelialization is an essential process during wound healing. Fibroblast cells play a principal role in the wound repair process for re-epithelization of the injured tissue [26]. It has been reported that proliferation and migration of keratinocytes from the edges of the wound for the re-epithelialization process depend on the interaction of keratinocytes with fibroblasts and the skin matrix [27]. In our data, MWO induced fibroblast-to-myofibroblast differentiation by acquiring *α*-SMA expression and the enhanced expression of the skin matrix including collagen as important events during wound healing (Figure 2 and Figure 5). Furthermore, in animal experiments, MWO significantly increased the re-epithelialization after skin injury (Figure 7). Thus, we think that MWO affects the role of interactions among keratinocytes, fibroblasts and myofibroblasts.

## 4. Materials and Methods

### 4.1. Materials

Antibodies against the phosphorylated form or total form of VEGFR-2, ERK, AKT and p38 were purchased from Cell Signaling Technology (Danvers, MA, USA). Antibodies against glyceraldehyde 3-phosphate dehydrogenase (GAPDH), horseradish peroxide (HRP) and conjugated secondary antibodies were supplied by Santa Cruz Biotechnology (Santa Cruz, CA, USA).

### 4.2. Cell Culture

NIH-3T3 fibroblast cells were purchased from American Type Culture Collection (Manassas, VA, USA) and cultured in DMEM (Dulbecco’s modification of Eagle medium; WELGENE, Gyeongsan, Korea) with *L*-glutamine (200 mg/L), 10% (*v/v*) heat-inactivated fetal bovine serum(FBS; WELGENE, Gyeongsan, Korea) and antibiotics (100 U/mL penicillin and 100 μg/mL streptomycin (Thermo Fisher Scientific, Waltham, MA, USA). Human umbilical vein endothelial cells (HUVEC) were purchased from ScienCell Research Laboratories, Inc. (Carlsbad, CA, USA) and cultured in endothelial growth medium-2 (ECM), also sourced from ScienCell Research Laboratories, Inc. (Carlsbad, CA, USA) with fetal bovine serum (FBS), endothelial cell growth supplement (ECGS) and penicillin/streptomycin solution (P/S). All cell lines were incubated at 37 °C in a humidified 5% CO_2_ cell culture incubator.

### 4.3. The Preparation of MWO

Mealworm, purchased from Yeochoun Bugs Land (Yeochoun, Pyeongtaek, Korea), was dried for 6 min using a 1000 watt microwave oven. To obtain mealworm oil, 1 kg of dried mealworm was pressed using a pressing machine (Poonggin Inc., Pyeongtaek, Korea) and filtered with cotton mesh. The samples were stored in a freezer at −70 °C until analysis.

### 4.4. Cell Viability

The cytotoxic effect of MWO was evaluated by using the methylthiazolyldiphenyl-tetrazolium bromide assay (MTT assay, Sigma-Aldrich, St. Louis, MO, USA). The cells were incubated in 24-well plates with the indicated concentrations of MWO for 24 h. Then, MTT solution (0.5 mg/mL) was added to each well. After incubation for 3 h at 37 °C in a CO_2_ incubator, the formazan crystals were dissolved in 400 μL of ethanol–(dimethyl sulfoxide) DMSO (*v/v*, 1:1). The cytotoxicity was estimated by measuring the absorbance at 540 nm with a microplate reader (Spectramax M2; Molecular Devices). The results from three independent experiments are presented as means ± SD. * *p* < 0.05, ** *p* < 0.01, and *** *p* < 0.001 indicate significance compared to the control.

### 4.5. Reverse-Transcription Polymerase Chain Reaction (RT-PCR)

Total RNA was isolated from NIH-3T3 and HUVEC cells using RiboEx^TM^ (GeneAll, Seoul, Korea). Equal amounts of total RNA (0.5 μg) from each sample were then subjected to reverse transcription with oligo-dT primers by using Moloney Murine Leukemia Virus (M-MLV) reverse transcriptase (Enzynomics, Daejeon, Korea). The cDNA was amplified by PCR using DiaStar^TM^ Taq DNA Polymerase (Solgent Co., Daejeon, Korea). The primers and PCR conditions used for amplifying the transforming growth factor beta-1 (TGF-β1), α-SMA, COL-1, VEGF and *GAPDH* are shown in Table 1.

### 4.6. Tube Forming Assay

To check the capillary-like tube formation of HUVECs, these were cultured in Matrigel-coated 24-well plates [28] using the conditioned media (CM) harvested from NIH-3T3 cells treated with or without MWO. For the preparation of CM, the NIH-3T3 fibroblast cells (1 × 10^5^) seeded in 6-well plates were cultured in 1.5 mL of DMEM serum-free medium with MWO—for 24 h. After incubation, 1 mL of the CM was harvested and mixed with ECM serum-free medium at a 1:1 ratio. The HUVECs were incubated with the mixed CM in matrigel-coated 24-well plates. After 12 h incubation, the tube formation of each well was photographed with a Nikon (ECLIPSE, TS100, Melville, NY, USA) light microscope.

### 4.7. Wound Healing Assay

For the wound healing assay using fibroblast cells, NIH-3T3 (1 × 10^6^ cells) were seeded and incubated in a 24-well plate. After the wells were fully filled with the cells forming a monolayer, wound gaps were created by scratching with a SPLScar^TM^ (SPL, Pyeongtaek, Korea). The cells were treated with MWO. After 24 h of incubation, the wound fields that migrated from either side of the cells were photographed with a Nikon (ECLIPSE, TS100) light microscope. To check the wound healing of HUVECs, the wound HUVECs were cultured using the CM harvested from NIH-3T3 cells treated with or without MWO. For the preparation of CM, the NIH-3T3 fibroblast cells (1 × 10^5^) seeded in 6-well plates were cultured in 1.5 mL of DMEM serum-free medium with MWO for 24 h. After incubation, 1 mL of the CM was harvested and mixed with ECM serum-free medium at a 1:1 ratio. HUVECs (1 × 10^5^ cells) were seeded and incubated in a 24-well plate. After the wells were fully filled with cells as the monolayer, wound gaps were created by scratching with a SPLScar^TM^ (SPL, Pyeongtaek, Korea). HUVECs were incubated with the mixed CM. After 12 h of incubation, the wound fields that migrated from either side of the cells were photographed with a Nikon (ECLIPSE, TS100) light microscope.

### 4.8. Western Blot Analysis

Total protein from HUVECs was extracted using 1% NP-40 lysis buffer (150 mM NaCl, 10 mM HEPES (pH 7.45), 1% NP-40, 5 mM sodium pyrophosphate (Na_4_P_2_O_7_), 5 mM sodium fluoride (NaF), 2 mM sodium orthovanadate (Na_3_VO_4_) containing protease inhibitor cocktail tablet (Roche, Mannheim, Germany). Equal amounts (30 μg) of proteins were used for the Western blot analysis. To detect target proteins, membranes were incubated with a 1:1000 dilution of primary antibodies against each protein and reacted with the corresponding HRP-conjugated secondary antibodies. Bands were detected with Pierce ECL (ThermoFischer Scientific, Waltham, MA, USA) and using ImageQuant LAS4000 (GE Healthcare, Pittsburgh, PA, USA).

### 4.9. Animal Experimental Design

Animal experiments were conducted in accordance with the ethical standards of the Animal Experimentation Ethics Committee of the College of Medicine of Yeungnam University (YUMC-AEC2019-005). Five male Sprague-Dawley (SD) rats (4 groups, *n* = 5) aged eight weeks and weighing 250 to 350 g were used in the experiment. For adaptation to the experimental environment, the rats were raised for one week in the living conditions that were used throughout the duration of the experiment (e.g., temperature, humidity, light and air). Food and drinking water were freely supplied. Sprague Dawley rats was anesthetized by intra peritoneal injection of 20 mg/kg ketamine and 10 mg/kg xylazine. The back area hairs of the rats were shaved, then sterilized with 70% ethanol. Four full-thickness circular wounds (6 mm diameter) were made on the dorsa skin at the back of the rats. The wound sites were treated twice a day with the extracted MWO at concentrations of 0, 10 and 100 μL. After three days, the wound sites were excised. Subsequently, their cellular structures, the expression of epidermal protein, neoangiogenetic cell markers (cluster of differentiation 31, CD31) and pancytokeratin were investigated by histological and immunohistochemical evaluation.

### 4.10. Histological Analysis

The areas where the skin defects had been made were harvested, including areas of normal tissue, and the specimens were fixed in formalin. Later, they were cut into sections with a thickness of 3 μm using a microtome. Then, after each specimen was attached to silane-coated slides, they were subjected to deparaffinization and hydration. Hematoxylin-eosin (H & E) staining and Masson trichrome staining were carried out. Tissue slides obtained by Masson trichrome staining were quantitatively evaluated for collagen fiber deposition using a computerized image analyzer (Leopard; Zootos, Uiwang, Korea).

### 4.11. Immunohistochemistry

Immunohistochemistry was performed using DAKO EnVision™+ System (Agilent Dako, Santa Clara, CA, USA). Briefly—formalin-fixed, paraffin-embedded tissue sections of 3 μM thickness were deparaffinized and rehydrated. Heat-induced epitope retrieval was performed using citrate buffer (pH 6.0) in a pressure cooker (IHC world, LLC., steamer set 120 v, 60 Hz, 650 w) for 45 min. The endogenous peroxidase activity was blocked using 3% H_2_O_2_ in methanol for 10 min. Tissue sections were then incubated for 60 min with rabbit polyclonal anti-CD31 (1:100, Bioss, Woburn, MA, USA) and Cytokeratin (1:100, Bioss, Woburn, MA, USA) at room temperature (25 °C) in a humidified chamber. After three washes for 5 min with Tris-buffered saline (TBS), tissue slides were incubated with EnVision + System-HRP Labelled Polymer Anti-Rabbit as per the manufacturer’s instructions (Agilent Dako, Santa Clara, CA, USA). Tissue sections were incubated with 3,3′-diaminobenzidine/H_2_O_2_ (Agilent Dako, Santa Clara, CA, USA) for color development and counter stained with Mayer’s hematoxylin. The stained tissue slides were dehydrated, cleaned and mounted in synthetic mountant (Thermo Scientific, USA). Microscopic photographs were taken with a DP70 microscopic digital camera (Olympus, Japan). Images acquired of five areas were randomly selected at high power resolution (×200) and photographed, and the number of blood vessels per unit area was assessed as a mean value using a computerized image analyzer (Leopard; Zootos, Uiwang, Korea). After immunohistochemical staining against pan-cytokeratin, Digital Slide Images were scanned with an Aperio CS2 Digital Slide Scanner (Leica Biosystems, Buffalo Grove, IL, USA). Re-epithelialization length was measured by Aperio ImageScope—Pathology Slide Viewing Software (Leica Biosystems, Buffalo Grove, IL, USA).

### 4.12. Statistical Analysis

The values from assessments of cell viability, gene expressions using RT-PCR, tube formation, migration, and Western blot analysis and data from the rat experiments were calculated by the percentage of control cells or fold induction and are expressed as the mean ± SD. The differences between the mean values compared with the control groups were evaluated by one-way analysis of variance with a post hoc Dunnet’s comparison using GraphPad Prism software (GraphPad Software, La Jolla, CA, USA). The minimum level of significance was set at a *p* value of 0.05 for all analyses.

## 5. Conclusions

In conclusion, in the present study, we confirmed that MWO promotes wound repair of skin in both in vitro and in vivo models. Particularly in the wound healing process, MWO induces the myofibroblast differentiation of fibroblast cells and collagen expression in fibroblast cells. The enhanced expression of VEGF in fibroblasts by MWO affects the increments of migration and tube formation of endothelial cells. These effects might then promote collagen deposition, angiogenesis and re-epithelialization at the sites of skin wounds. Thus, we believe that MWO might have potential as a therapeutic agent for the treatment of skin wounds.

## Figures and Tables

**Table 1:** Primers used for PCR of TGF-β1, α-SMA, COL-1, VEGF and GAPDH.

Gene	Primer Sequences	PCR Condition	Size (bp)
*mTGF-β1*	Forward: 5′-AGGAGACGGAATACAGGGCT-3′ Reverse: 5′-CCACGTAGTAGACGATGGGC-3′	60 °C 30 cycles	482
*mα-SMA*	Forward: 5′-CTGACAGAGGCACCACTGAA-3′ Reverse: 5′-CATCTCCAGAGTCCAGCACA-3′	55 °C 30 cycles	160
*mCOL-1*	Forward: 5′-AAGAGGCGAGAGAGGTTTCC-3′ Reverse: 5′-AGAACCATCAGCACCTTTGG-3′	55 °C 25 cycles	244
*mVEGF*	Forward: 5′-CAGCACATAGGAGAGATGAGC-3′ Reverse: 5′-TCACCGCCTCGGCTTGTCACA-3′	60 °C cycles	234, 306
*mGAPDH*	Forward: 5′-AACTTTGGCATTGTGGAAGG-3′ Reverse: 5′-ACACATTGGGGGTAGGAACA-3′	60 °C 30 cycles	223

## Data Availability

The data that support the findings of this study are available from the corresponding author upon reasonable request.

## Sample Availability

Samples of compounds are available from the authors.
